# Associations between ideational variables and bed net use in Madagascar, Mali, and Nigeria

**DOI:** 10.1186/s12889-018-5372-2

**Published:** 2018-04-11

**Authors:** J. Douglas Storey, Stella O. Babalola, Emily E. Ricotta, Kathleen A. Fox, Michael Toso, Nan Lewicky, Hannah Koenker

**Affiliations:** Johns Hopkins Center for Communication Programs, 111 Market Place, Suite 310, Baltimore, MD 21202 USA

**Keywords:** Malaria, Ideation, Bed nets, Insecticide treated nets, Health communication, Behavior change

## Abstract

**Background:**

The use of insecticide-treated bed nets (ITNs) is crucial to the prevention, control, and elimination of malaria. Using household surveys conducted in 2014–2015 by the Health Communication Capacity Collaborative project in Madagascar, Mali, and Nigeria, we compared a model of psychosocial influence, called Ideation, to examine how malaria-related variables influence individual and household bed net use in each of these countries. Evaluations of non-malaria programs have confirmed the value of the ideational approach, but it is infrequently used to guide malaria interventions. The study objective was to examine how well this model could identify potentially effective malaria prevention approaches in different contexts.

**Methods:**

Sampling and survey designs were similar across countries. A multi-stage random sampling process selected female caregivers with at least one child under 5 years of age for interviews. Additional data were collected from household heads about bed net use and other characteristics of household members. The caregiver survey measured psychosocial variables that were subjected to bivariate and multivariate analysis to identify significant ideational variables related to bed net use.

**Results:**

In all three countries, children and adolescents over five were less likely to sleep under a net compared to children under five (OR = 0.441 in Madagascar, 0.332 in Mali, 0.502 in Nigeria). Adults were less likely to sleep under a net compared to children under five in Mali (OR = 0.374) and Nigeria (OR = 0.448), but not Madagascar. In all countries, the odds of bed net use were lower in larger compared to smaller households (OR = 0.452 in Madagascar and OR = 0.529 in Nigeria for households with 5 or 6 members compared to those with less than 5; and OR = 0.831 in Mali for larger compared to smaller households). Of 14 common ideational variables examined in this study, six were significant predictors in Madagascar (all positive), three in Mali (all positive), and two in Nigeria (both negative).

**Conclusion:**

This research suggests that the systematic use of this model to identify relevant ideational variables in a particular setting can guide the development of communication strategies and messaging, thereby improving the effectiveness of malaria prevention and control.

## Background

As of 2016, sub-Saharan Africa carries the vast majority of the global burden of malaria cases (90%) and deaths (92%); 70% of the deaths were in children under five [1]. The use of ITNs has been shown to reduce malaria incidence rates by 50% in children under five [1] and mass ITN distribution campaigns and routine distribution channels have been shown to be effective and cost-efficient ways of increasing household ITN access and reducing malaria morbidity and mortality [2–7]. However, while access to nets remains a barrier to achieving consistent net use in some places [4, 8–10], the question persists of how to ensure that people obtain and use the nets to which they have access.

At a systems level, access to bed nets is ensured by a country’s ability to provide bed nets through mass or continuous distribution, and retail or social marketing channels to achieve universal coverage [4]. The standard Roll Back Malaria indicator of population access to ITN refers to the proportion of individuals that could use a net within a household, assuming one net covers two people. An additional standard indicator is the proportion of households that own enough ITNs to achieve the World Health Organization’s universal coverage recommendation of one ITN for every two people [4, 8, 9, 11]. Recent reports indicate that ITN use and access are strongly correlated [10, 12]. Over several recent MIS and DHS surveys, Madagascar and Mali both have very high ratios of ITN use to ITN access (> 1.00, indicating more than 2 people on average share an ITN, and > 0.90, respectively), while Nigeria’s 2015 MIS documented a ratio of 0.68. In all three countries, the ratio varies at the subnational level and by socioeconomic status and residence [10]. In the few countries where access and use are not strongly correlated, and among groups of people with access who do not use ITNs, social and behavior change communication (SBCC) campaigns offer the most feasible means of closing the ITN access/use gap [10, 12].

SBCC campaigns can help to change or reinforce behaviors necessary to obtain, and/or maintain the appropriate number of ITNs in a household, as well as sleep under a bed net [4, 13–16] by focusing on particular psychosocial determinants of behavior. Most behaviors are not driven by a single variable or determinant. For example, preventive health behaviors do not result from a fear of disease alone, but are also—often simultaneously—influenced by such things as concerns about the cost or inconvenience of protective measures, confidence or doubts about the effectiveness of a treatment, and motivation to do what others in the community do [17]. In this study, we conducted a comparison of a particular model of psychosocial influence, known as the Ideation Model of Strategic Communication and Behavior Change [4, 18–20], to examine how malaria-related ideational variables influence individual and household bed net use in each of these countries. Evaluations of programs on other health topics have confirmed the predictive value of the ideational approach, but it has not been widely used to guide malaria interventions. The main objective of this study was to determine the degree to which the ideation model was generalizable as a guide to identifying potentially effective malaria prevention approaches in three different countries.

**Fig. 1:**
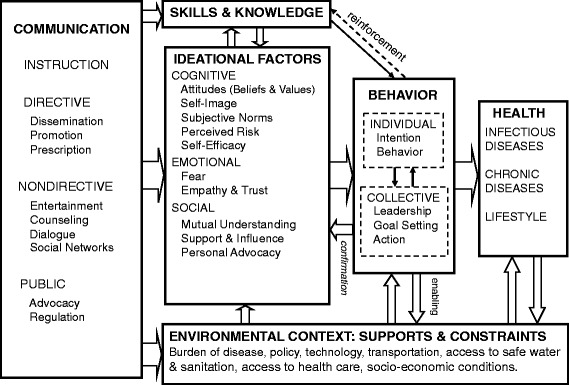
Ideation model of strategic communication and behavior change. Detailed legend: Ideation model of strategic communication and behavior change showing relationships between types of communication, skills and knowledge, environmental context, ideation, behavior change and health outcomes [73]

The ideation model (Fig. 1) is part of a metatheory of strategic communication and behavior change [19, 20] that incorporates intermediate cognitive, emotional and social constructs from various behavioral theories and models. Different theories tend to emphasize different factors and variables associated with behavior change. Some emphasize cognitive variables, such as beliefs, values, and attitudes [21–25]; others include emotional variables, such as fear or elation, empathy, and confidence, or self-efficacy [26–28]; while others place more emphasis on conative variables, such as social support, social influence, spousal/partner communication, and personal advocacy [29–35]. These are listed in the central box of Fig. 1. While many SBCC campaign strategies emphasize social and psychological determinants of behavior, the ideation model described here is unique in that it emphasizes three things: (1) an individual’s decision-making process leading to a behavioral choice is complex and can involve multiple variables simultaneously, (2) ideational variables are behavior-specific, and (3) the influence of those multiple variables is cumulative, that is, the more of the variables that are positive with regard to the behavior, the higher the probability of that behavior occurring. Furthermore, all ideational variables can be influenced by social interaction and a variety of instructive, directive, non-directive and public communication forms, often through mass media exposure [4, 11, 36, 37], which increases the likelihood of population-level change.

Figure 1 also indicates that the ideational change resulting from communication complements other changes in skills and knowledge necessary to perform a particular action, like bed net use, as well as changes in the environmental context, like socio-economic conditions, policies and types of material and technological support for health improvement efforts. When ideational change occurs and is supported by appropriate knowledge and environmental conditions, the behavior of individuals and groups of people is likely to change, resulting in improved health outcomes. The more ideational factors that improve, the greater the probability of behavior change. This model has been used to help guide and explain the effects of a variety of health behavior programs, including contraceptive adoption [36, 38–40], HIV testing [41], HIV prevention [42], child survival [43], household treatment and handling of water [44, 45], community support to reduce girls’ vulnerability to HIV/AIDS [46], and Ebola response [47]. Human-centered design approaches [48] in health and other behavioral domains have begun to use the ideation model to understand customer needs and develop client-oriented products that resonate with those needs.

In the case of malaria, one would not expect the same set of beliefs, attitudes, norms, and perceptions to influence, for example, both bed net use and care seeking for fever. Similarly, one would not expect the same set of ideational variables to influence bed net use both in year-round malaria areas and in areas with highly seasonal transmission, because patterns of risk perception and susceptibility are likely to be quite different, thus underscoring the importance of exploring the ideational variables that are relevant for each behavior and in different settings.

Recently in Tanzania, the first malaria-related study to focus on the ideation process found that a particular set of ideational variables mediated the effects of malaria prevention communication campaigns on household-level universal coverage with ITNs [4]. Ideation about bed net use in Tanzania included the following locally specific variables: (1) perceived positive social norms surrounding net ownership and use, (2) belief in one’s ability to use nets properly (self-efficacy), (3) belief that net use is an effective way to prevent malaria (sometimes termed ‘response’ efficacy), and (4) perceived threat of malaria. Exposure to communication campaigns was correlated positively with increased net ideation, that is, the cumulative number of positive ideational variables, which in turn was correlated positively and significantly with household universal ITN coverage, i.e., having enough nets for everyone in the household [4].

The Health Communication Capacity Collaborative (HC3) conducted household surveys in Madagascar, Mali, and Nigeria, to examine the relationships between an individual’s net-use behavior and their demographic and psychosocial (ideational) variables. In this article, we compare the ideational variables of bed net use to prevent malaria in these three countries in order to better understand how differences in ideation can be used to inform strategies to improve the effectiveness of malaria prevention and control interventions wherever bed net use is a public health priority.

## Methods

### Study setting

This study is based on analysis of household survey data collected in Madagascar, Mali, and Nigeria between 2014 and 2015 by the Johns Hopkins Center for Communication Programs (CCP), under the United States Agency for International Development (USAID)-funded HC3 project and with the support of the President’s Malaria Initiative (PMI). The surveys were designed specifically for the purpose of comparing ideational influences on the use of bed nets for malaria prevention as part of CCP’s mandate under this grant. These countries were chosen for the study because they are PMI priority countries.

CCP designed the questionnaires, but field work was conducted by trained interviewers through a contract with a private research firm in each country, selected through competitive procurement. Questionnaires were translated from the original English into local languages. Depending on respondents’ preferences, the interviews were conducted in English, Pidgin English, Igbo or Hausa in Nigeria; French or Bambara in Mali; and Malagasy in Madagascar. Data were collected during the dry season/early rainy season in Madagascar (between September and November 2014), the rainy season in Mali (between July and September 2015), and the rainy season in Nigeria (between July and September 2015).

The three countries have differing levels of malaria parasitemia in children 6–59 months—measured through microscopy—varying from 9% in Madagascar [49] to 27% in Nigeria [50] and 36% in Mali [51]. While malaria transmission in Nigeria is stable and perennial, northern Sahalian areas experience increases in transmission during 3 months or less of seasonal rains [52]. Mali’s Sahalian belt experiences stable, seasonal transmission while in the northern desert region transmission is low, unstable and epidemic-prone [53]. In Madagascar, the sub-desert and highland zones experience shorter seasons of increased transmission, the latter with pockets of high elevation where autochthonous transmission is rare; the tropical and equatorial zones experience stable malaria transmission most of the year [54]. Cyclones occur between December and April and often result in increased risk of malaria infection. The majority of malaria cases are caused by *Plasmodium falciparum* parasites in all three countries.

Mass bed net distributions took place in each of the three countries prior to survey data collection. While sensitization campaigns accompany most mass distributions, details about SBCC coverage, message exposure, and message recall are only available for a program in Nigeria, namely the Support to the National Malaria Programme (SuNMaP) funded by UKaid, and the NetWorks project funded by the U.S. President’s Malaria Initiative [55]. In that instance, over half of respondents in ten different states were exposed to multi-channel SBCC and positive attitudes towards nets were positively associated with the number of messages recalled.

### Sampling design and participant characteristics

The sampling design was similar across the three surveys. Study participants were selected through a multi-stage random sampling process that involved first selecting—with probability proportional to size—districts, local government areas, or communes depending on the country, then clusters or enumeration areas, and finally households with at least one child under 5 years old. In each selected household, a child under 5 years old was randomly selected and the mother of that child was invited to participate in the caregiver survey. In our experience, household heads and caregivers are able to describe not only their own behavior, but that of other members of the family with a fairly high level of reliability. This is especially true for observable behaviors like sleeping under a bed net. We anticipated little additional informational value from multiple interviews in the same household, so opted to interview the caregiver of only one under-five child in each household and distribute the sample across a wider variety of households. In addition, in one third of the selected households, the head of household (if he/she was not the same as the caregiver) was also interviewed to obtain the perspective of the key decision-maker in the household. The multistage sampling design was taken into consideration in calculating the sample size. We applied a design effect of 2.0. Sample weights were not available; so, they were not used in the analyses. The sampling strategy yielded 2390 households in Madagascar, 3202 in Mali, and 3616 in Nigeria. Using a structured household survey questionnaire, information on ownership and use of bed nets was collected on all household members: 12,834 in Madagascar, 19,345 in Mali, and 16,832 in Nigeria. In this manuscript, we merged household-level data with data from the caregiver survey to assess the correlates of household members’ net use. Specifically, from the household data, we derived bed net use information and sociodemographic characteristics of individual household members, while data on female caregivers’ sociodemographic and ideational variables were derived from the individual caregiver questionnaire.

### Variables

In this study, the dependent variable was whether an individual slept under a bed net on the night before the survey, as reported in the household questionnaire. The variable was derived from a question that asked who slept under each net available, as enumerated in a household net roster, following the Roll Back Malaria Monitoring and Evaluation Working Group’s recommendations [56]. Net brand was not recorded consistently, therefore determining a net’s status as an ITN was not possible; however, the majority of bed nets in each country are ITNs as observed in recent surveys—94% in Madagascar in 2013, 97% in Nigeria in 2015, and 95% in Mali in 2015 [49–51]. We assessed the predictive value of 25 independent variables measured at the individual, household, and community levels, including household size and the number of nets owned, and the following female caregiver-specific variables:

- education level and religion;
- radio-listening and television-viewing habits;
- exposure to malaria related-information on media or through community sources;
- perceived severity of malaria*;
- perceived susceptibility to malaria*;
- perceived self-efficacy to prevent malaria*, to detect a severe case of malaria*, or to procure enough nets for all members of her household*;
- perceived response efficacy of bed nets*;
- knowledge of fever as a symptom of malaria and of mosquitoes as the cause of malaria*;
- discussion of malaria with friends and family during the last 12 months*;
- participation in decisions about net allocation within the household*;
- attitudes toward bed nets*;
- perception about bed net use being the norm in their community*;
- awareness of a place to purchase nets*; and
- willingness to pay for nets*.

A cumulative ideation score was then calculated as the sum of the number of ideational variables that are positively associated with bed net use in each country (among the items with * above), producing an ideation score.

In addition to the main models that included the individual ideational variables, other models that substituted a composite core—the sum of the number of positive individual ideational variables for each respondent—were tested to assess the Sp: cumulative predictive power of ideation. In addition, we calculated an interaction term between age and sex in the estimated models for all three countries.

### Analysis

We used both bivariate and multivariate analytic methods. The bivariate method compares net use across sociodemographic groups and reports the significance of the differences. Logistic regression was the main multivariable analytic method used and was limited to individuals from households with at least one net and at least one child under 5 years old, representing 73% of households in Madagascar, 97% in Mali, and 79% in Nigeria. We report both the odds ratios and the fully-standardized beta coefficients for the regression models.

## Results

In this section, we report the analysis results for each country separately, then summarize findings across all three countries in the discussion section.

### Madagascar

#### Bivariate analysis

Overall, almost three-quarters (73.3%) of residents of households with at least one net slept under a net. Among these households, net use varied significantly by zone of residence: 39.4% in the Highlands compared to 71.4% in Sub-Desert, 72.9% in Tropical, and 82.3% in Equatorial (X^2^ = 447.3; *p* < 0.001). Significant differences by age were identified: 79.9% of children under 5 years old slept under a net compared to 54.5% among children aged 5–17 years, and 80.1% of adults (X^2^ = 469.8; *p* < 0.001). Significant differences by gender were also found, with net use being more common among females (75.5%) than among males (70.9%) (z = 4.95; *p* < 0.001).

#### Multivariate analysis

Results of the multivariate logistic regression indicate that the significant demographic predictors of net use among household members are the individual’s age, zone of residence, household wealth, household size, and number of nets in the household. Also significant were the female caregiver’s television viewing and her level of exposure to malaria-related communication messages. There were no significant interactions between age and gender (Table 1).

**Table 1:** Results of logistic regression of household members’ net use by selected sociodemographic, ideational, and household variables in Madagascar, Mali, and Nigeria

Predictor	Madagascar		Mali		Nigeria	
	Odds Ratio	Beta^a^	Odds Ratio	Beta^a^	Odds Ratio	Beta^a^
Age group						
Under-5 (RC)	1.00	–	1.00	–	1.00	–
5–17	0.411***	− 0.192	0.332***	− 0.265	0.502***	−0.162
Adult	0.934	−0.016	0.374***	−0.242	0.448***	−0.202
Gender						
Male (RC)	1.00	–	1.00	–	1.00	–
Female	1.155	0.034	0.839‡	−0.044	1.044	0.011
Age group/Gender Interactions						
Under-5 X Male (RC)	1.00	–	1.00	–	1.00	–
5–17 X Female	0.978	− 0.004	1.459***	0.073	1.084	0.015
Adult X Female	1.282‡	0.049	3.296***	0.247	1.667***	0.107
Household wealth quintile						
Lowest (RC)	1.00	–	1.00	–	1.00	–
Second	0.971	−0.006	0.913	−0.019	0.960	−0.008
Middle	0.849‡	−0.029	0.852*	−0.031	0.962	−0.008
Fourth	0.801**	−0.041	0.711***	−0.065	0.950	−0.011
Highest	0.989	−0.002	0.646***	−0.088	0.879‡	−0.026
Household size^b^			0.831***	−0.316		
Household size^b^						
2–4 (RC)	1.000	–				
5–6	0.452***	−0.173				
7–8	0.246***	−0.274				
9 +	0.147***	−0.338				
Household size^b^						
2–4 (RC)					1.00	1.00
5–6					0.529***	−0.150
7–9					0.365***	−0.234
10 +					0.207***	−0.313
Number of nets in household^c^						
1–2 (RC)			1.00	–		
3–4			2.722***	0.240		
5 or more			5.931***	0.415		
Number of nets in household^c^						
1–2 (RC)	1.00	–				
3 or more	2.793***	0.218				
Number of nets in household^c^						
0–1 (RC)					1.00	–
2					0.893***	−0.028
3 or more					0.646***	−0.100
Zone of residence						
Highlands (RC)	1.00	–				
Sub-desert	3.595***	0.278				
Tropical	2.928***	0.231				
Equatorial	5.076***	0.352				
Region of residence						
Koulikoro			1.00	–		
Sikasso			1.787***	0.130		
Mopti			1.231***	0.045		
Bamako			0.952	−0.010		
State of residence						
Akwa Ibom (RC)					1.00	–
Kebbi					1.487***	0.098
Nasarawa					1.099‡	0.022
Caregiver’s education						
Primary or less (RC)	1.00	–	1.00	–	1.00	–
Post-primary	0.925	−0.011	0.849**	−0.032	0.957	−0.010
Caregiver’s religion						
Non-Christian (RC)	1.00	–			1.00	–
Christian	0.888‡	−0.026			0.865*	−0.037
Caregiver’s radio listening habits						
Fewer than once a week (RC)	1.00	–	1.00	–	1.00	–
At least once a week	0.911	−0.022	0.977	−0.006	1.024	0.006
Caregiver’s TV watching habits						
Fewer than once a week (RC)	1.00	–	1.00	–	1.00	–
At least once a week	1.348**	0.041	1.227***	0.051	1.034	0.008
Caregiver’s exposure to messages on malaria						
No exposure (RC)	1.00	–	1.00	–		
Low	0.975	−0.006	0.962	−0.009		
High	1.447***	0.073	0.899	−0.018		
Heard messages on malaria					0.960	−0.010
Caregiver’s perceived severity of malaria						
Lower (RC)	1.00	–	1.00	–	1.00	–
Higher	0.971	−0.006	0.973	−0.007	0.885**	−0.031
Caregiver’s perceived susceptibility to malaria		–				–
Lower (RC)	1.00		1.00	–	1.00	
Higher	0.998	−0.000	0.977	− 0.006	0.927‡	0.019
Caregiver’s perceived self-efficacy to prevent malaria						
Lower (RC)	1.00	–	1.00	–	1.00	–
Higher	1.567***	0.061	1.068	0.016	1.034	0.008
Caregiver’s perceived self-efficacy to detect a serious case of malaria						
Lower (RC)	1.00	–	1.00	–	1.00	–
Higher	1.162**	0.033	0.940	−0.014	0.986	−0.003
Caregiver’s perceived response-efficacy of nets						
Lower (RC)	1.00	–	1.00	–	1.00	–
Higher	1.137	0.017	1.060	0.012	0.919*	−0.021
Caregiver’s awareness that fever is a symptom of malaria						
Not aware (RC)	1.00	–	1.00	–		
Aware	0.985	−0.003	1.155***	0.035		
Caregiver’s awareness that mosquito is the cause of malaria						
Not aware (RC)	1.00	1.00	1.00	–	1.00	–
Aware	0.982	−0.003	1.365***	0.048	0.933	−0.010
Caregiver’s perceived self-efficacy to purchase enough nets						
Lower (RC)	1.00	–	1.00	–		
Higher	1.344***	0.067	1.200***	0.044		
Discussed malaria with friends/relations	1.057	0.013			0.996	−0.001
Participates in decisions about net allocation	1.343***	0.061			0.998	−0.001
Attitudes towards nets						
Negative (RC)	1.00	–			1.00	–
Positive	1.020	0.005			0.968	−0.008
Perceived net use as the norm among in community	1.391***	0.069			1.024	0.006
Knows where to buy nets	1.284***	0.058			1.023	0.005
Willing to pay for nets	1.015	0.003			1.045	0.011
Pseudo-R^2^	17.1%		9.9%		8.6%	
Hosmer-Lemeshow GOF^d^ (***X***^2^/ p)	15.3/0.08		15.5/0.08		14.9/0.09	
Number of Observations	9260		19,345		15,463	

Notes: ‡ *p* < 0.1; * *p* < 0.05; ** *p* < 0.01; *** *p* < 0.001

^a^Fully (XY) standardized beta coefficients

^b^Household size was not normally distributed in Madagascar and Nigeria. The variable was differently categorized in the two countries in a way that helps to ensure model fit

^c^Number of nets was not normally distributed in any of the study countries. The variable was differently categorized in the three countries in a way that helps to ensure model fit

^d^*GOF* Goodness of Fit

Among males, household members aged 5–17 years were 59% less likely than children under 5 years old to sleep under a net. There was no significant difference in net use between adult males and male children under 5 years old. Among children under 5 years old, gender did not make a difference in the odds of net use. Additionally, being female did not significantly moderate the relationship between age and net use. The relationship of net use and household wealth was such that the only significant difference was found between those in the lowest quintile and their peers in the fourth quintile. The relationship between net use and household size was negative. For example, people from households with nine or more members were 85% less likely than those from households with fewer than five members. Furthermore, having three or more nets in the household increased the odds of net use almost three-fold compare to having one or two nets.

Whereas female caregiver’s regular radio listenership (at least once a week) made no difference in individual member’s net use by 15%, regular television viewership (at least once a week) increased the odds by 35%. The female caregiver’s low exposure to malaria-related communication messages (one or two messages recalled) did not make a difference compared to no exposure; however, a high level of exposure (three or more messages recalled) was associated with a 45% percent higher likelihood of household member’s net use.

Significant ideational variables for female caregivers included perceived self-efficacy to prevent malaria, perceived self-efficacy to detect malaria, perceived self-efficacy to obtain enough nets for members of her household, perceived response efficacy of nets, descriptive norm about nets, awareness about where to procure nets, and level of participation in decisions regarding net allocation within the household. Residents of households where the female caregiver demonstrated perceived self-efficacy to prevent malaria were 57% more likely to sleep under a net than those in households where the female caregiver did not demonstrate such belief. Similarly, the female caregiver’s self-efficacy to detect a severe case of malaria was associated with a 16% increase in the odds of household member use of a net while the caregiver’s perceived self-efficacy to obtain enough nets for her household was associated with a 34% increase in the odds. Female caregiver’s belief that net use was the norm in her community increased the odds of household member’s net use by 39% while her awareness of where to procure a bed net increased the odds by 28%. Finally, members of a household in which the female caregiver participates in net allocation decisions were 34% more likely to sleep under a net than those in households where the woman did not participate in such decisions.

A look at the magnitude of the fully standardized beta coefficients reveals that the most important predictors of household members’ net use were the individual zone of residence, number of nets in the household, and household size and age, in order of importance. Among the female caregiver’s ideational variables, the most important were descriptive norms (perceptions about how other people behave), perceived self-efficacy to procure enough nets for the household, participation in household net allocation decisions, perceived self-efficacy to prevent malaria, and awareness of where to procure nets.

**Fig. 2:**
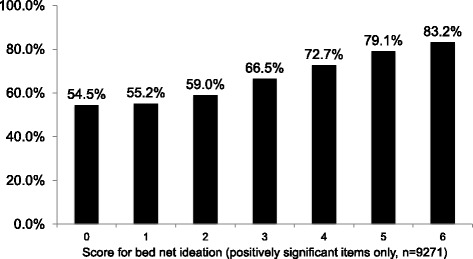
Predicted probability of household bed net use, by female caregiver’s cumulative ideation score, Madagascar 2014. Detailed legend: Probability of household bed net use as a function of female caregivers’ cumulative ideation score in Madagascar 2014 (range = 0–6, mean = 4.13, SD = 1.18)

Figure 2 shows a graduated, dose-response relationship of net use with ideation score, controlling for all other non-ideational variables. The percent who slept under a net on the night before the survey increased from 54.5% in households where the female caregiver had zero positive ideational variables (out of six) to 83.2% in households where the female caregiver had all six positive ideational variables. When the composite ideation score (mean = 4.13, SD = 1.18) was substituted for the individual ideational variables in a logistic regression, the results indicate a strong positive relationship, controlling for all other non-ideational variables. Specifically, a one-point increase in the caregiver’s ideation score increases the household member’s odds of sleeping under a net by about 37%.

### Mali

#### Bivariate analysis

In Mali, possession of nets was almost universal in 2014–2015: only 3.1% of the households in the study regions did not have at least one bed net. Net use information was available for 19,345 household members (9436 males and 9909 females). The majority of household members (82.1%) slept under a net on the night preceding the survey. Differences by gender, age group, and region of residence were significant. More than four-fifths (85.3%) of females compared to 78.7% of males slept under a net on the night before the survey (z = 14.2; *p* < 0.001). Net use was less prevalent among older children aged 5–17 years (76.2%) compared to children under five (88.4%) and adults (84.1%) (X^2^ = 298.3; *p* < 0.001). Variations by region of residence were also noticeable, varying from 78.5% in Koulikoro to 87.3% in Sikasso (X^2^ = 155.4; *p* < 0.001).

#### Multivariate analysis

Results of the multivariate logistic regression revealed that the predictors of net use in Mali operate at various levels and include age, gender, number of nets in the household, and region of residence (Table 1). Other significant demographic predictors include household wealth, household size, and level of education of the female caregiver.

Among males, the odds of net use were significantly lower among boys aged 5–17 years (67% lower) and men (63% lower) compared to children under five. Among children under five, there was no difference between boys and girls in the odds of net use. The association of age with net use depends on gender: being female attenuates the negative relationship of bed net use with age. There was a graduated reduction in the odds of net use across wealth quintiles such that the odds of net use were about 35% less in households in the highest quintile and 15% less in households in the middle quintile compared to the poorest households. The relationship of net use with household size was negative: each additional household member decreased the odds of net use by 17%. In contrast, the relationship with the number of nets was positive: people in households with three or four nets were almost three times as likely and those in households with five or more nets were almost six times as likely to use a net compared to their peers in households with one or two nets. Whereas there was no difference between Koulikoro and Bamako, the residents of Sikasso and Mopti were significantly more likely to use a net compared to their peers in Koulikoro.

The association of net use with caregiver’s education was negative in Mali, with members of households where the woman has post-primary education being about 15% less likely to use a net compared to their peers in households where the woman had primary education or less. Female caregiver’s regular radio listening was not associated with net use, but regular television viewing increased the odds of household members’ net use by 23%. The caregiver’s exposure to malaria-related messages made no significant difference in net use independently of what was attributable to general media habits.

In terms of ideational variables, there was a strong positive association with caregiver’s awareness of fever as a symptom of malaria and mosquito as the cause of malaria. Similarly, the relationship with caregiver’s perceived self-efficacy to obtain enough nets for members of her family was positive and significant. Rather surprisingly, household members’ net use was negatively associated with the female caregiver’s perceived self-efficacy to detect a severe case of malaria.

Examination of fully standardized beta coefficients revealed that the most important determinants of household members’ net use were age, household size, and number of nets. The most important caregiver’s ideational variables were her malaria-related knowledge and her perceived self-efficacy to obtain enough nets for members of her family.

The multivariable logistic regression that included the overall ideation score (mean = 2.73, SD = 1.08) in place of the individual ideational variables revealed a dose response relationship between caregiver’s ideation score and household members’ net use. For example, the odds of household member net use was more than twice (2.06) as high in households where the female caregiver scored three out of three on the positive ideation score compared to households where the female caregiver scored zero out of three.

### Nigeria

#### Bivariate analysis

In Nigeria, only one-third (33.7%) of residents of households with at least one net slept under a net. Among these households, net use varied significantly by state of residence: 32.1% in Akwa Ibom compared to 38.2% in Kebbi and 29.9% in Nasarawa (X^2^ = 92.6; *p* < 0.001). There were also significant differences by age: 44.7% of children under 5 years old slept under a net compared to 24.4% among children age 5–17 years and 32.9% of adults (X^2^ = 463.3; *p* < 0.001). There were also significant differences by gender with net use being more common among females (36.2%) than males (31.1%) (X^2^ = 48,1; *p* < 0.001).

#### Multivariate analysis

Results of the multivariate logistic regression indicate that the significant predictors of net use among household members are household size, age, state of residence, number of nets, and being of Christian faith, in order of magnitude. Among males, compared to children under five, 5–17 year olds (50.2% lower) and adults (55.2% lower) were significantly less likely to have slept under a net. There was no significant sex difference among children under five. When looking at the interaction between age and gender, being female significantly improved the odds of adult net use. Unsurprisingly, as household size increased, odds of using a net decreased. For example, the likelihood of net use was about 79% lower for people from households with ten or more members compare to those from households with fewer than five members. There was a negative dose response association between net use and number of nets (a 12% decrease in likelihood), which is opposite of what we found in Madagascar and Mali. The odds of using a net were significantly higher in Kebbi (49%), but not in Nasarawa or Akwa Ibom. Respondents of Christian faith were 15% less likely to use a net than those practicing other religions.

Use of radio or television and reported level of exposure to malaria messages by the female caregiver were not significant predictors of net use in Nigeria. In terms of ideational variables, there was a negative relationship between net use and caregiver’s perceived severity of malaria, with the odds of net use being 12% lower among members of households where the caregiver has a higher level of perceived severity of malaria compared to their peers in household where the caregiver has a lower level of perceived severity. There was also a negative relationship between caregiver’s perceived response efficacy of nets and household members’ net use. No other ideational variables were significant in Nigeria.

## Discussion

The ideation model of communication and behavior change has been used effectively in a number of health domains to guide the strategic design and evaluation of health communication interventions, but it has received relatively little attention in malaria prevention efforts. This study attempted to compare the dynamics of ideation and behavior in three countries in order to determine how it differs by context. Results indicate that a more diverse set of ideational factors play a role in household bed net use in Madagascar compared to Mali or Nigeria, and that the strongest ideational predictors of bed net use vary from one country to another, although efficacy-related variables appear to be important in all three settings. In our view, this demonstrates the usefulness of ideation as a conceptual tool that can help researchers and program planners to identify common psychosocial variables and systematically examine how they influence malaria-related behavior in a particular programmatic context, controlling for prevailing local conditions.

### The role of independent demographic variables

In terms of demographic predictors of bed net use, there were more similarities than differences across the three countries analyzed in this study, even though Madagascar and Mali had much higher rates of bed net use (73% and 82%, respectively) compared to Nigeria (34%). In all three countries, children under 5 years old were more likely to sleep under a bed net compared to older children aged 5–17 years (and adults, except in Madagascar). Also in all three countries, the odds of bed net use were lower on average in larger households. The documented relationships with age and household size are consistent with what other studies have found in Africa and elsewhere [14, 57–60]. Also in Madagascar and Mali, bed net use was higher in the poorest households compared to wealthier households. Only in Mali were families whose caregivers had lower levels of education significantly more likely to use bed nets than families whose caregivers had more education. In Nigeria, Christians were less likely to use bed nets than Muslims. Findings of this nature can help programmers to target subgroups that are at higher risk.

In Madagascar and Mali, households with caregivers who regularly watched television were more likely to report bed net use. Consistent with findings from other studies, in Madagascar, caregivers’ higher malaria message recall was associated with higher rates of bed net use [4, 14, 16, 61]. The reasons for the lack of significant association between caregiver’s exposure to malaria messages and bed net use in Mali and Nigeria were not clear; we know little about the intensity or content of malaria messages except in Nigeria, so it is hard to attribute the findings to the reach or quality of the information provided. The overall lower use of bed nets in Nigeria may result in lower salience of malaria messaging, which would reduce motivations to pay attention to and process malaria information, even though communication campaigns were underway.

### Effects of independent ideational variables

In terms of ideational predictors of bed net use, multiple factors were positively correlated with net use in Mali and Madagascar. Counterintuitively, in Nigeria, the higher the caregiver’s perceived severity of a malaria infection or her perceived response efficacy of nets (i.e., belief that bed net use can prevent infection), the lower the reported bed net use. Some conceptual frameworks that focus on threat and efficacy as predictors of behavior see high perceived risk as potentially debilitating—a frightened person may not take action to counter a disease threat if there is no accompanying belief in one’s ability to manage the threat [27, 62–64]. Sometimes self-efficacy can result in lower rates of behavior if it takes the form of overconfidence to control threats and achieve health outcomes [65–68]. This may be the case in Nigeria, where there was no evidence that self-efficacy perceptions—self-efficacy to detect or prevent malaria or self-efficacy to obtain bed nets—were associated with bed net use. People may feel so confident in their ability to detect and treat a severe case of malaria that they feel less threatened by the disease, and are therefore less motivated to sleep under a net.

In Mali, consistent with findings from many previous studies, knowledge about malaria played a role in motivating behavior: people who knew that fever is a symptom of malaria and who knew that mosquitoes spread malaria were more likely to report bed net use [57, 69, 70]. This finding was in contrast to what we found for the other two study countries. Without more information about existing malaria communication in Mali, it is difficult to know why these differences exist. Some malaria programs in Sub-Saharan Africa emphasis rapid careseeking in response to fever as part of large scale “test and treat” campaigns. Such programs would draw attention to information about malarial fever caused by mosquitos versus non-malarial fever and the importance of being tested to know the difference. In such a case, a stronger association between mosquitos and fever could result in greater interest in bed net use to prevent both. Future analysis of how malaria campaigns with different emphases may reinforce (or interfere with) each other would be useful.

In Madagascar, self-efficacy to prevent malaria and self-efficacy to obtain bed nets were associated with higher reported net use, echoing findings from other studies, including a similar study conducted in Liberia [14]. Unlike Mali, in Madagascar self-efficacy to detect a severe case of malaria was positively associated with net use. Also in Madagascar three additional ideational variables were positive predictors of bed net use: the belief that bed net use was the community norm, knowing where to obtain a bed net, and participation at the household level in decision-making about who would sleep under a bed net. Why more ideational variables were significant in Madagascar compared to Mali or Nigeria is not clear. Greater familiarity or experience with an issue is often associated with greater cognitive elaboration; if malaria communication (whatever its content) is more intensive or far-reaching in one place compared to another, that could explain more nuanced perceptions about disease threat and prevention. On the other hand, where exposure to information has been low, the introduction of an information campaign can have novelty effects—people learn a lot very quickly—whereas familiarity with an issue sometimes results in more counterarguing and rejection of new information. More research on the history and content of malaria communication in these countries would be needed to determine which explanation is more likely.

As summarized in Table 2, of the 14 ideational variables examined in this study, six were significant predictors of bed net use in Madagascar (all positively), four were significant predictors of bed net use in Mali (all positively), and only two were significant predictors in Nigeria (both negatively).

**Table 2:** Summary of ideational predictors of bed net use, by country

Predictor	Country		
	Madagascar	Mali	Nigeria
Perceived severity of a malaria infection	ns	ns	–
Self-efficacy to detect severe case of malaria	+	ns	ns
Self-efficacy to prevent malaria	+	ns	ns
Self-efficacy to obtain enough nets for the household	+	+	ns
Response efficacy (belief that nets are effective)	ns	ns	–
Perceived community norm of bed net use	+	na	ns
Knowledge of where to get bed nets	+	na	ns
Knowledge that fever is a sign of malaria	ns	+	ns
Knowledge that mosquitos are the malaria vector	ns	+	ns
Participating in decision-making about net allocation	+	na	ns
Number of positively significant ideational predictors of bed net use	7	3	0

Key: + (positively relationship), − (negatively significant), *ns* (not significant), *na* (not measured)

Madagascar and Mali share only one of the ideational predictors: self-efficacy to obtain enough bed nets for your household. In Nigeria, perceived severity of malaria and perceived response efficacy of nets were the only ideational variables associated (negatively) with bed net use. This finding suggests that structural and demographic barriers as well as psychosocial influences may need to be addressed as determinants of bed net use in that country.

It is important to note that another study in Nigeria that included measures of female caretakers’ malaria-related ideation [71] found it to be significantly associated with the caretaker’s own use of a bed net, rather than bed net use by members of her household. Other factors may also explain why household bed net use is more weakly correlated with caretaker’s ideation in Nigeria. For example, it may be that female caregivers in Nigeria have less agency to influence bed net use by other members of the household compared to female caregivers in Mali and Madagascar, thus weakening the causal link between caregiver ideation and household member behavior.

### Implications for effective malaria social and behavior change strategies

It is essential to note that age, region, household size, and number of nets in the household were the strongest predictors of bed net use, demonstrating that families are prioritizing younger children, consistent with recent studies [72], and that the primary barriers to bed net use are still largely due to access. Controlling for these structural barriers permits SBCC programs to then isolate pertinent ideational variables. Of all the ideational variables examined in these three countries, those that were the most consistently significant predictors of bed net use were related to efficacy perceptions: self-efficacy to obtain bed nets for your household and self-efficacy to detect malaria. This suggests that efficacy-based communication strategies are likely to produce desirable results. Programs should emphasize increasing and reinforcing the head of household’s and caregiver’s confidence in responding to the threat of malaria and their correct and consistent net use as a preventive measure. The widely validated social learning approach of modeling effective behavior [21] could be used to show or describe locally acceptable and identifiable examples of effective net use and other preventive behaviors in an effort to build local self-confidence in malaria prevention. Showing how nets can be obtained and used may be particularly important in places like Nigeria where net use and the access to use ratio are low to begin with, and might complement expanded distribution efforts and accelerate uptake. Finally, while we cannot confirm the relationship between female agency and decision making using the current datasets, it may be important in settings where caregivers have less household authority to influence decision makers or other female caregivers in the same household. In such settings, modeling efficacious communication in the household about bed net use could be beneficial.

This study also provides support for the hypothesis of a cumulative impact of ideational variables. In Madagascar and Mali, where multiple ideational variables were significant predictors of bed net use (controlling for the effect of household and sociodemographic variables), bed net use was higher when more ideational variables were positive. This suggests that it may be effective to bundle more than one ideational variable into program messaging, for example, by addressing multiple aspects of self-efficacy and malaria knowledge in an integrated communication strategy.

### Limitations of this study

The use of bed nets by household members was determined through an enumeration of household residents, of the nets available in the household, and through subsequent questions about who slept under each of those nets on the night prior to the survey. This produced an outcome variable representing whether an individual slept under a net the night before the survey. Yet, data on ideational variables were collected only from the female caregivers of a selected child under 5 years old in each household. Our analysis assumes that what a particular female caregiver—who may be only one of several caregivers in the household—knows, thinks, and feels about malaria and bed net use will have an impact on the bed net use patterns of the entire household. Ultimately, bed net use is a volitional behavior, particularly for older children and adults who may have the power to make their own choices and are therefore influenced by their own individual attitudes, beliefs, and preferences as opposed to those of the female caregiver who was interviewed. While our data indicate that there is a positive relationship between female caregiver ideation and household net use, controlling for numerous other household and demographic variables, the actual causal pathways are undoubtedly more complicated. Future studies may wish to collect ideational data from more than one individual in a household or, perhaps, include more questions about the content of interpersonal discussion of malaria prevention and net use among household members, in order to better understand the broader household context of net use decisions.

## Conclusion

While the strongest predictors of net use among households with at least one bed net were age, region, household size, and number of nets, this study has demonstrated the importance of controlling for these variables when trying to identify the ideational variables that influence caregiver bed net use in three African countries, because the relevant ideational variables varied across countries. In Madagascar, the two most significant ideational predictors of bed net use were the caregiver’s descriptive norm about bed net use and her awareness of where to procure a bed net. In Mali, the most important caregiver’s ideational variables were her perceived self-efficacy for obtaining bed nets and her malaria-related knowledge. While no ideational variable was positively linked with bed net use in Nigeria, this finding calls for additional study to identify other possible social, structural, and supply-side explanations for bed net use.

In short, this research suggests that integrating more than one ideational variable—such as malaria knowledge and one or more aspects of self-efficacy—into program messaging, could result in a more effective integrated communication strategy.

## Abbreviations

HC3: Health Communication Capacity Collaborative
IRB: Institutional Review Board
ITN: insecticide-treated bed net
PMI: U.S. President’s Malaria Initiative
SBCC: social and behavior change communication
USAID: United States Agency for International Development
